# Uncovering potential molecular biomarkers for cancer-associated secondary lymphedema through integrated analyses of RNA-sequencing, machine learning, and clinical data

**DOI:** 10.3389/fonc.2026.1760040

**Published:** 2026-02-13

**Authors:** Hao Dong, Jianliang Miao, Zhong Liu, Yuguang Sun, Peilin Li, Song Xia, Wenbin Shen

**Affiliations:** 1 Department of Lymphatic Surgery, Capital Medical University Affiliated Beijing Shijitan Hospital, Beijing, China; 2 Clinical Center for Lymphatic Disorders, Capital Medical University (CMU), Beijing, China; 3 First Affiliated Hospital of Dalian Medical University, Dalian Medical University, Dalian, China

**Keywords:** immune infiltration, machine learning, ScRNA, secondary lymphedema, SHAP

## Abstract

**Background:**

Cancer-associated secondary lymphedema (CASL) commonly occurs after tumor-related lymph node dissection and radiotherapy. Nevertheless, the mechanisms of CASL remain unclear, and there are no specific molecular markers for its diagnosis and treatment.

**Methods:**

In this study, RNA sequencing was performed on adipose tissues from 10 normal controls and 40 patients with CASL. Differentially expressed genes were screened using two machine learning algorithms to identify potential molecular markers for CASL. Subsequently, seven machine learning algorithms were employed to develop predictive models based on the identified markers. The contribution of each feature to the predictive outcomes was interpreted using Shapley additive explanation (SHAP). Immune cell infiltration was profiled through CIBERSORT and MCP-counter algorithms, and single-cell RNA sequencing (scRNA-seq) data were integrated to explore interactions between characteristic genes and immune cell subpopulations. Furthermore, associations between characteristic genes and clinical parameters were also assessed.

**Results:**

IL2RG, HOXD10, and TSPAN1 were identified as potential biomarkers of CASL. Diagnostic models built on these three genes showed excellent performance. Functional enrichment analysis suggested that the dysregulation of cytokine-cytokine receptor interactions and immune pathways underlies the pathological progression of CASL. In addition, immune infiltration analysis indicated that T cell and macrophage infiltration were intricately involved in CASL progression. Intriguingly, single-cell transcriptomic analysis further revealed elevated expression of IL2RG, HOXD10, and TSPAN1 in T cell subsets. Finally, RT-qPCR validated that these genes were expressed at higher levels in CASL tissues than in normal tissues. Moreover, IL2RG expression was strongly associated with clinical parameters.

**Conclusions:**

This study identified IL2RG, HOXD10, and TSPAN1 as novel potential molecular markers for CASL, providing valuable biological evidence for the diagnosis and intervention of CASL.

## 1 Introduction

Lymphedema is the abnormal accumulation of lymph fluid in interstitial spaces resulting from lymphatic system dysfunction. It is classified into primary and secondary forms based on etiology. Primary lymphedema is usually caused by congenital defects, whereas secondary lymphedema (SL) is mainly induced by external factors such as surgical intervention, radiotherapy, and infection. Globally, most cases of lymphedema are secondary. CASL is the most common cause of SL and predominantly occurs after tumor-related lymph node dissection and radiotherapy (1, 2), particularly following surgeries for gynecological and breast malignancies (3). The incidence of lower limb lymphedema among gynecological cancer survivors has been reported to range from 0% to 70% (4). In one study, 292 patients (32.4%) out of 900 older women with endometrial, colorectal, or ovarian cancer reported CASL; prevalence was highest in ovarian cancer survivors (36.5%), followed by endometrial cancer survivors (32.5%) (5). Among breast cancer survivors, over one-fifth may develop upper limb lymphedema (6).

The primary clinical manifestations of CASL include chronic limb swelling, a sense of heaviness, pain, recurrent infections, and decreased quality of life. Although the molecular mechanisms remain incompletely understood, CASL has been associated with inflammatory factors, CD4+ T cell and macrophage infiltration, lymphedema-associated fibroblasts (LAFs), microRNAs, and other elements. At present, CASL is diagnosed mainly by imaging and interventional examinations, which can be invasive and difficult to implement broadly. There are still challenges in developing new approaches based on molecular biomarkers (7). Current therapies include manual therapy, compression therapy, complete decongestive therapy (CDT), and surgery. However, no specific drugs are available for CASL, and the development of molecular therapies is still ongoing. Owing to the complexities of diagnosis and treatment, as well as the prolonged duration of the disease, CASL imposes significant economic and psychological burdens on patients (8–11). Therefore, exploring molecular biomarkers associated with the pathogenesis of CASL will aid in developing new diagnostic and intervention strategies to enhance prognosis and quality of life for CASL patients.

High-throughput sequencing technologies facilitate rapid and efficient genome-wide analysis, elucidating gene expression patterns across diverse pathological states to identify potential molecular markers (12, 13). Machine learning is increasingly used for biomarker discovery in transcriptomics and proteomics. By applying algorithms such as least absolute shrinkage and selection operator (LASSO) regression, random forest, and support vector machine-recursive feature elimination (SVM-RFE), key features that reflect biological differences can be extracted. Furthermore, advanced computational frameworks support robust model training and evaluation through systematic data partitioning and cross-validation. To date, the combination of high-throughput sequencing (RNA-seq) and machine learning has been widely adopted to identify disease-specific genes and to construct diagnostic and prognostic models (14, 15). Although previous studies have utilized RNA-seq to explore pathogenic genes for CASL (3), no research integrated RNA-seq with machine learning algorithms to find distinctive biomarkers for CASL.

In this study, we integrated RNA-seq and machine learning analyses to identify potential molecular markers for CASL. Two feature-selection algorithms (LASSO and SVM-RFE) were implemented on RNA-seq data to determine core genes, which were subsequently utilized to build diagnostic models via seven different machine learning methods. SHAP was further leveraged to interpret the contribution of each feature to model predictions. These algorithms possess inherent advantages that diminish data dimensionality while maintaining model robustness, thereby yielding more reliable outcomes. Additionally, we employed the CIBERSORT and MCP-counter algorithms to characterize immune cell infiltration in CASL and incorporated scRNA-seq data to further investigate the crosstalk between signature genes and immune cell subpopulations.

## 2 Materials and methods

### 2.1 Study design and participant selection

**Figure 1:**
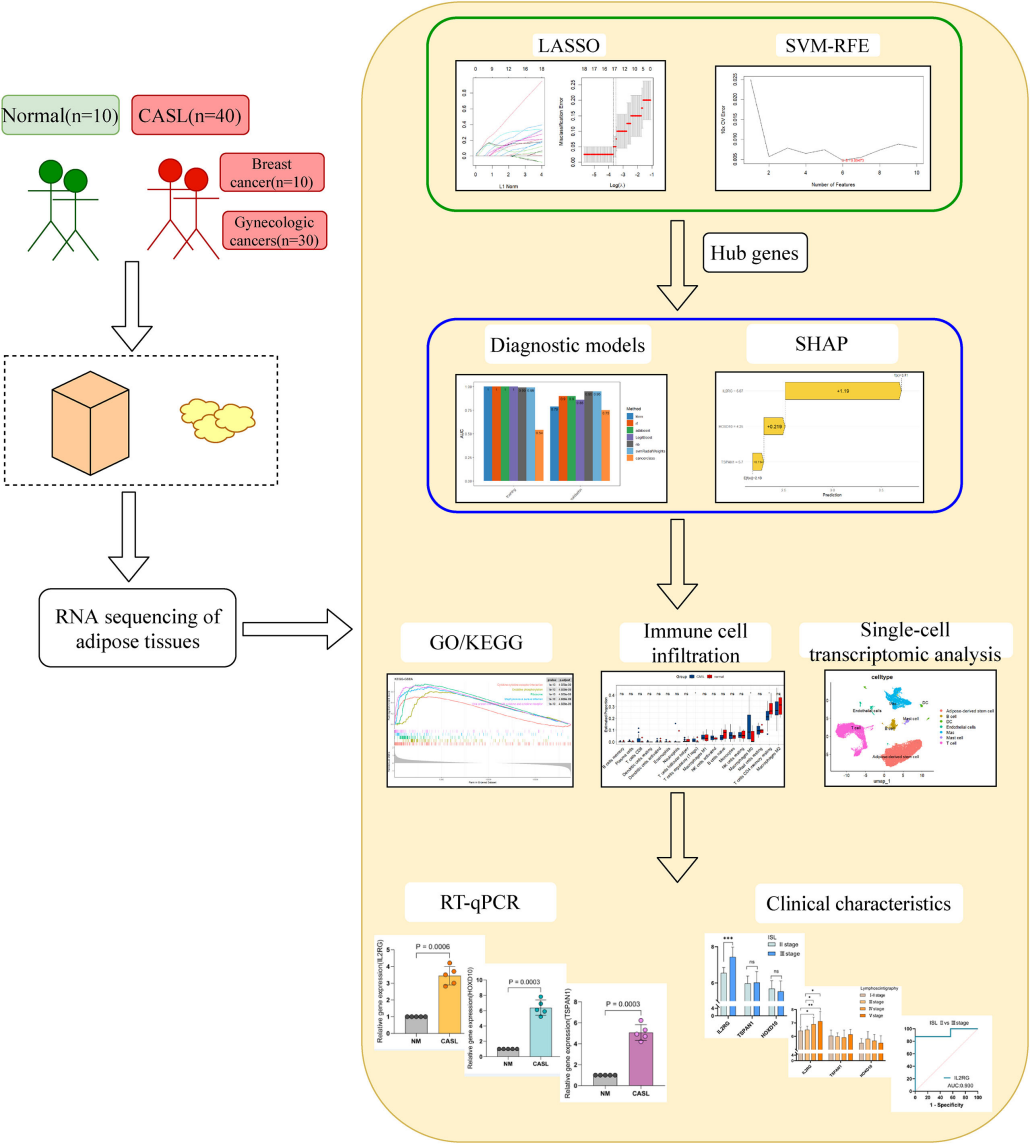
The overall process of this study.

The overall process of this study is illustrated in **Figure 1**. RNA-seq was performed on adipose tissue samples from 10 healthy controls and 40 patients with CASL. The protocol was approved by the Ethics Committee of Beijing Shijitan Hospital affiliated with Capital Medical University and complied with the Declaration of Helsinki. Inclusion criteria for CASL patients were: (1) clinically diagnosed with cancer-related secondary lymphedema, meeting the diagnostic criteria of the International Society of Lymphology (ISL) (16); (2) aged 18 years or older; (3) no prior surgical treatment related to CASL (such as liposuction, lymphatic venostomy, lymph node transplantation, etc.). Exclusion criteria included: (1) the presence of systemic diseases that can cause swelling (such as heart disease, kidney disease, digestive tract disease, venous insufficiency, etc.); (2) patients with recurrent or multiple cancers during the observation period; (3) prior surgical treatment related to CASL.

A total of 50 female participants were enrolled in this study, categorized into 40 patients with CASL and 10 healthy female volunteers who served as the normal control group. The CASL group comprised 10 cases of breast cancer-related lymphedema and 30 cases of gynecological cancer-related lymphedema. Pathological adipose specimens were collected from the affected limbs of patients with CASL, whereas normal adipose samples were obtained from the limbs of healthy individuals. Baseline characteristics were comparable between the two groups. Specifically, the mean age was 57.58 ± 7.53 years in the CASL group and 57.0 ± 6.87 years in the control group (P > 0.05). The mean BMI was 27.12 ± 4.62 kg/m² for the CASL group and 26.9 ± 3.50 kg/m² for the control group (P > 0.05). All participants were female. No statistically significant differences were observed in age or BMI between the groups, indicating well-matched baseline characteristics.

### 2.2 Collection of human samples and RNA extraction

Adipose tissue samples were rinsed with phosphate-buffered saline (PBS) and swiftly frozen in liquid nitrogen for subsequent RNA extraction. The TRIzol Reagent (Invitrogen, USA) was used to extract the RNA. To evaluate the extracted RNA’s quality and concentration, a NanoDrop 2000 spectrophotometer (Thermo Fisher Scientific, USA) was used. RNA integrity was evaluated with the RNA Nano 6000 Assay Kit on an Agilent 2100 Bioanalyzer (Agilent Technologies, USA), and only samples with an RNA integrity number (RIN) greater than 7.0 were considered suitable for further analysis.

### 2.3 RNA sequencing analysis

To construct the sequencing library, mRNA was isolated using poly-T oligo-linked magnetic beads, followed by cDNA synthesis, end repair, adapter ligation, and PCR amplification using the Illumina TruSeq RNA Sample Preparation Kit (Illumina, San Diego, USA). The quality of the resulting library was validated using an Agilent 2100 Bioanalyzer. High-throughput paired-end sequencing of 150 bp was conducted on the Illumina HiSeq 2500 platform.

### 2.4 Bioinformatics analysis

#### 2.4.1 Differentially expressed genes analysis

Differential expression gene analysis was performed to identify genes that differed between the CASL and control groups. By calculating the dynamic logFC values, we establish a threshold to select genes that might have notable alterations with biological significance. This process was implemented utilizing the R package “limma”. Furthermore, the calculation formula is as follows.

|logFC| > [mean (|logFC|) + 2sd (|logFC|)] (17).

The DEGs analysis thresholds were ultimately chosen at P < 0.05 and |logFC| >0.6.

#### 2.4.2 Enhancement of functionality

To investigate the biological functions of DEGs, Gene Ontology (GO) and Kyoto Encyclopedia of Genes and Genomes (KEGG) enrichment analyses were performed using the “clusterProfiler” R package.

#### 2.4.3 Hub genes selection

To identify characteristic markers of CASL, DEGs were subjected to feature selection using two algorithms: LASSO and SVM-RFE. As an established regularization approach, LASSO incorporates an L1 penalty into the loss function by summing the absolute values of model coefficients. This process induces sparsity by driving non-essential coefficients toward zero to enable autonomous feature selection. SVM-RFE operates by iteratively training SVM models and eliminating features with the least significance. In this study, these algorithms were implemented via the ‘glmnet’ and ‘e1071’ R packages, respectively. To enhance the robustness of the identified features, the results from both methods were intersected, and the overlapping candidates were ultimately defined as the hub genes associated with CASL.

#### 2.4.4 Construction of diagnostic models

The dataset was randomly partitioned into a training set and a validation set at a ratio of 7:3. Based on the hub genes, seven distinct machine learning classifiers were developed to establish diagnostic models. The Receiver Operating Characteristic (ROC) curve was applied to judge the performance of these predictions, and the algorithms’ predictive effectiveness was gauged by calculating the Area Under the Curve (AUC). These models were implemented using the R package “mime”. The specific configurations of the machine learning algorithms used in this study are summarized in **Supplementary Table S2**.

#### 2.4.5 SHAP analysis

To further highlight the significance of features and graphically illustrate how they affect model predictions in machine learning models, SHAP was implemented. SHAP analyses were performed via the R package “shapviz”.

#### 2.4.6 GSEA

To show the characteristics of gene expression in highly enriched functional pathways, Gene set enrichment analysis (GSEA) was performed. Two-sided p < 0.05 was considered statistically significant. GSEA is implemented through the R package “clusterProfiler”.

To explore the functional differences of individual genes in CASL, after identifying hub genes in the previous analysis, we conducted an in-depth analysis of the gene expression patterns found in the functional pathways connected to these crucial genes. In particular, CASL samples were stratified into high- and low-expression groups for each hub gene using the median expression value as the cutoff. GSEA was then performed to compare pathway enrichment between the high- and low-expression subgroups, enabling evaluation of the functional impact of each hub gene in CASL.

#### 2.4.7 Evaluation and correlation analysis of immune cells

After determining the relative abundances of immune cell populations based on the LM22 gene signature matrix provided by CIBERSORT, which was accomplished using the R package “cibersort”, the levels of various immune cell types in samples from CASL and healthy control participants were compared. Furthermore, to complement the analysis and obtain estimates of absolute cell abundances, we employed the Microenvironment Cell Populations-counter (MCP-counter) method. The analysis was performed using the R package “MCPcounter”, which calculates abundance scores based on cell-type-specific gene signatures. This method provides absolute quantification scores for eight immune cell lineages, as well as for two key stromal cell populations—fibroblasts and endothelial cells—offering a broader view of the tissue microenvironment. The resulting scores were then compared between the CASL and healthy control cohorts. Next, Spearman’s correlation analysis was subsequently performed to assess associations between hub gene expression and immune cell infiltration levels.

#### 2.4.8 ScRNA-seq data processing

All scRNA-seq data come from the 10X Genomics original count study published by Liu et al. (18). The scRNA-seq samples in this study were obtained from subcutaneous adipose tissue of the affected thigh in five female patients who developed secondary lymphedema following surgical treatment for cervical cancer (ISL stage III). To isolate the non-adipocyte fraction, the stromal vascular fraction (SVF) was obtained via collagenase I digestion of the adipose tissue. Freshly isolated SVF cells from each sample were processed separately for scRNA-seq on an Illumina NovaSeq 6000 system (Illumina, USA). For batch correction, data integration, and quality control, we used the Seurat and Harmony R packages for five single-cell samples. We removed genes detected in ≤5 cells and cells with expression counts below 2% or above 98%. Then, we normalized samples via log-normalization. Further, we used PCA to identify the top 30 principal components of the top 30 highly-variable genes. Clustering was done using FindNeighbors and FindClusters functions (resolution = 0.2), and all cells were split into 14 clusters using the UMAP algorithm. Each cluster was annotated with known cell-type-specific markers. Moreover, in Seurat, we used FindAllMarkers and FindMarkers functions to detect DEGs for each cell type based on the non-parametric Wilcoxon rank-sum test. Then, we combined DEGs with the CellMarker website to manually annotate cell types further.

#### 2.4.9 Hub gene score

Each cell was scored using the AUCell R package based on gene set enrichment. The AUC values of three hub genes were utilized to rank intra-cellular gene expression and estimate the proportion of highly expressed signatures. Cells with more genes in the gene set have higher AUC values. Determine the threshold for identifying gene - set - active cells with *AUCell_exploreThresholds* function. Finally, the resulting AUC scores were projected onto UMAP embeddings via the ggplot2 R package (version 3.3.5) to visualize active clusters.

### 2.5 Validation of hub genes by RT-qPCR in human samples

Real-time quantitative PCR (RT-qPCR) was performed to validate the expression of IL2RG, HOXD10, and TSPAN1 in adipose tissue from the control and CASL groups. Primer sequences are provided in **Supplementary Table S1**.

### 2.6 Analysis of hub genes in relation to clinical characteristics

The ISL, lymphoscintigraphic, and MRI stages were independently assessed by two senior lymphatic surgeons according to previously established criteria (19–21). Multi-frequency bioelectrical impedance analysis (MFBIA) data were acquired with an InBody 770 analyzer (Biospace, South Korea). Limb volume was calculated using the standard truncated cone formula (22).

### 2.7 Statistical analysis

Statistical analyses were conducted in R (version 4.4.1). Data were expressed as mean ± SD or median (IQR). Statistical analyses were performed using appropriate tests: Student’s t-test (for normally distributed data between two groups), Mann-Whitney U test or Kruskal-Wallis H test (for non-normally distributed data), one-way ANOVA (for multi-group comparisons), and Spearman’s correlation (for monotonic relationships). A two-sided *p* < 0.05 is considered the threshold for statistical significance.

## 3 Results

### 3.1 DEGs in CASL

**Figure 2:**
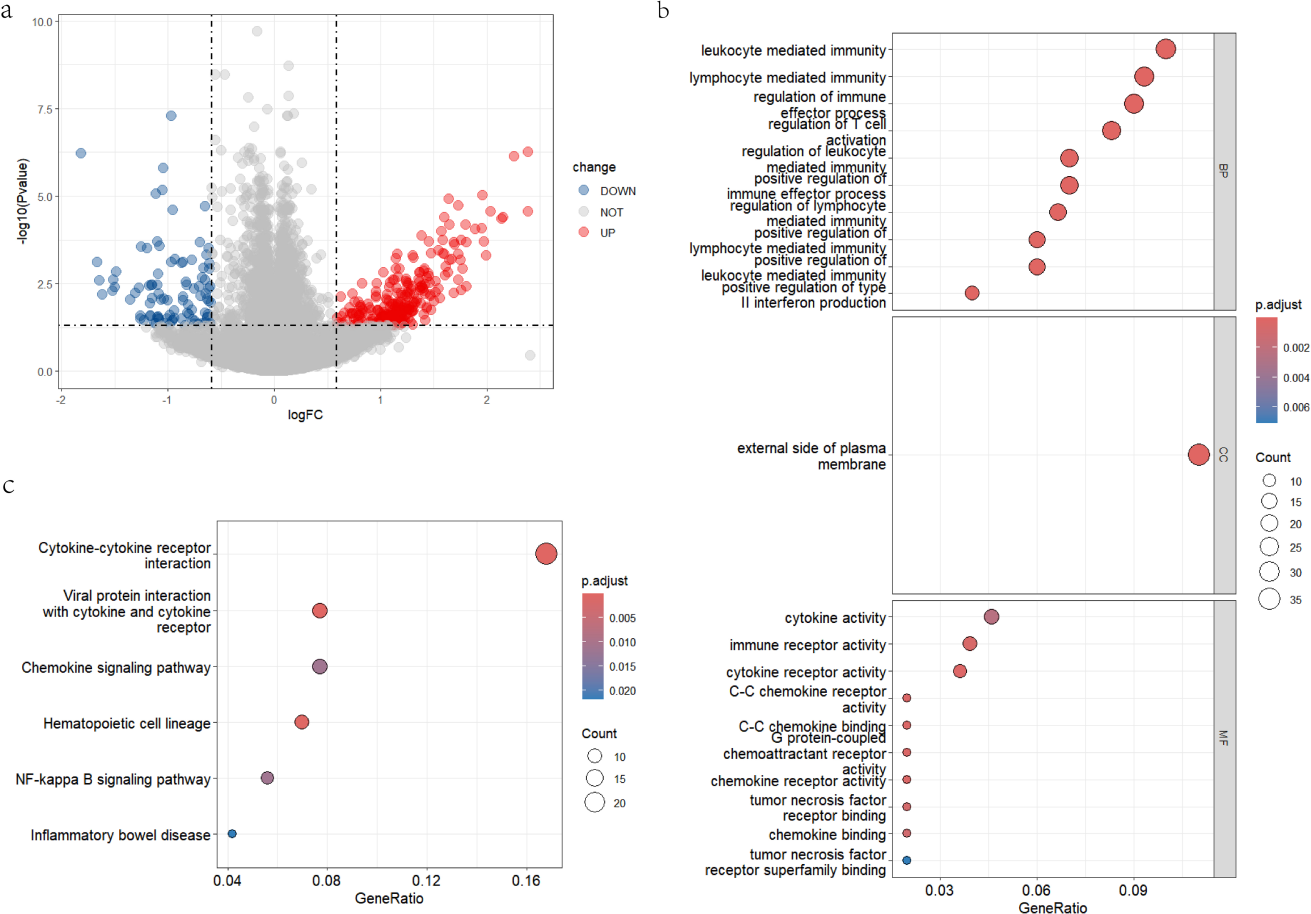
The identification and enrichment analyses of DEGs in CASL and normal control groups. **(A)** The volcano plot of DEGs. **(B)** Enrichment analysis of DEGs in GO. **(C)** Enrichment pathway analysis of DEGs in the KEGG database.

To characterize transcriptional differences between the CASL and control groups, differential expression analysis was performed using the R package “limma”. A total of 365 DEGs were identified, including 269 upregulated genes and 96 downregulated genes (**Figure 2A**).

### 3.2 Functional enrichment analysis of DEGs

To clarify functional differences between the CASL and normal groups and to gain mechanistic insight into the identified DEGs, GO and KEGG enrichment analyses were conducted. In the GO biological process category, DEGs were enriched in leukocyte mediated immunity, lymphocyte mediated immunity, regulation of immune effector process, regulation of T cell activation, and regulation of leukocyte mediated immunity. For cellular components, enriched terms were mainly related to the external side of the plasma membrane. In terms of molecular function, the main results included cytokine activity, immune receptor activity, and cytokine receptor activity (**Figure 2B**). KEGG pathway analysis revealed significant enrichment in cytokine-cytokine receptor interaction, viral protein interaction with cytokine and cytokine receptor, chemokine signaling pathway, hematopoietic cell lineage, and NF-kappa B signaling pathway (**Figure 2C**). Collectively, these results suggest that cytokine-receptor interactions and immune-related pathways may contribute to the pathological process of CASL.

### 3.3 Identification and validation of diagnostic genes for CASL through machine learning algorithms

**Figure 3:**
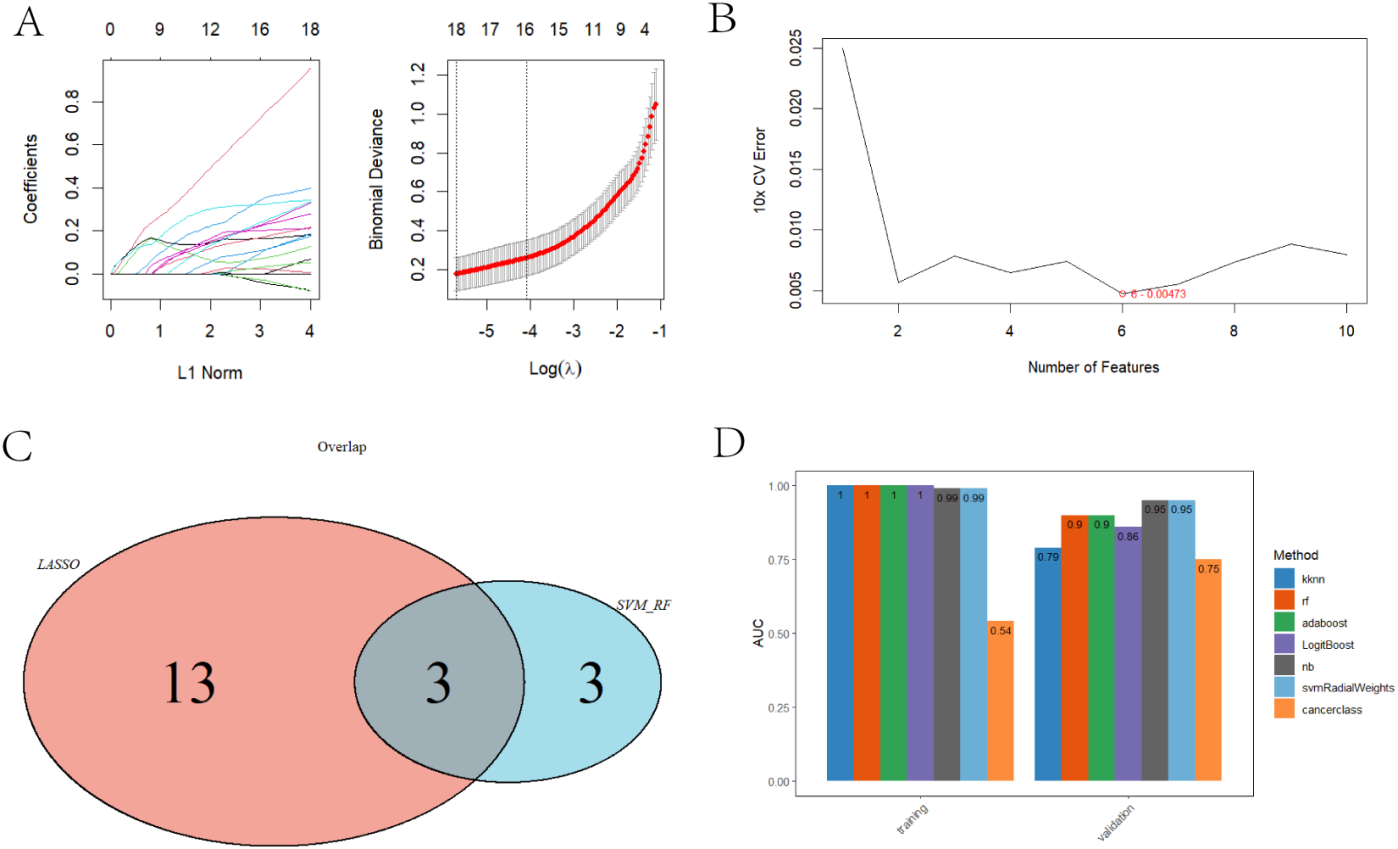
Screening and validation of hub genes by machine learning. **(A)** Feature selection based on LASSO. **(B)** Feature selection based on SVM-RFE. **(C)** The intersection of hub genes selected by LASSO and SVM-RFE. **(D)** ROC for the predictive models based on hub genes.

To identify the most discriminative hub genes between the CASL and normal groups, DEGs were further filtered using LASSO and SVM-RFE. In LASSO logistic regression, genes with non-zero coefficients are retained during model optimization, reflecting their contribution to classification. In this study, with the selected optimal regularization parameter (λ) of 0.004, LASSO identified a total of 16 non-zero coefficient genes that were crucial for disease prediction (**Figure 3A**). In the SVM-RFE process, the model evaluates all features according to gene weights, and gradually simplifies the model by recursively eliminating the least important features. During this process, the performance of the model was monitored through cross-validation. The 6 optimal genes with the lowest 10-fold cross-validation (CV) error were finally identified (**Figure 3B**). Subsequently, the intersection of these two methodologies yielded three signature genes (IL2RG, HOXD10, TSPAN1), which are proposed as potential diagnostic markers for CASL (**Figure 3C**).

**Figure 4:**
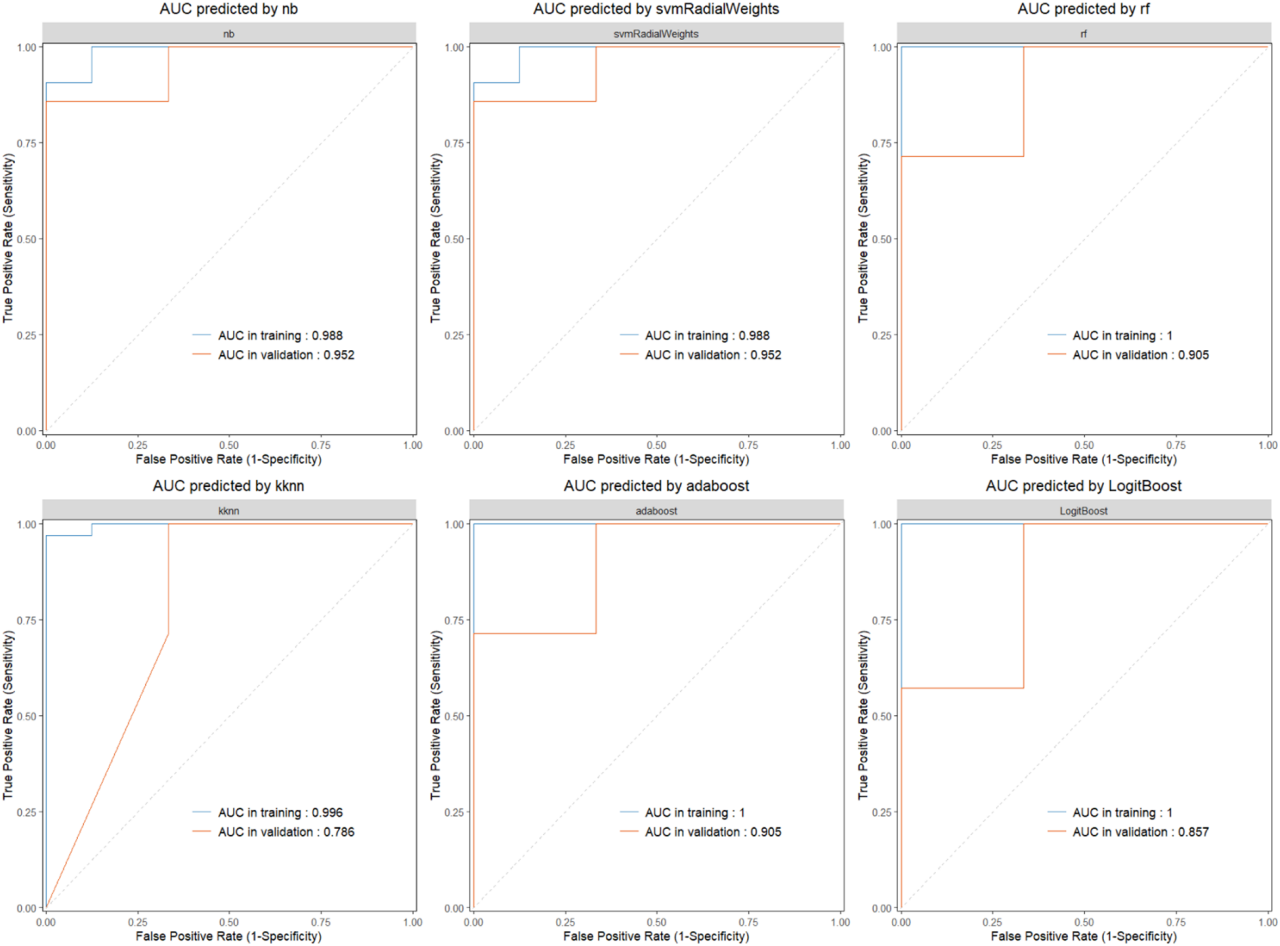
The ROC curve for predictive models built with ‘nb’, ‘svmRadialWeights’, ‘rf’, ‘kknn’, ‘adaboost’, ‘LogitBoost’.

To further evaluate the diagnostic utility of the three signature genes, we randomly divided the RNA-seq dataset into training and validation sets at a 7:3 ratio and constructed diagnostic models based on IL2RG, HOXD10, and TSPAN1 using seven machine learning algorithms (**Figure 3D**). In addition, ROC curves were generated to assess model discrimination, and AUC values were calculated to quantify predictive performance, with values closer to 1.0 indicating better accuracy. In the training set, the AUCs of the kknn, rf, adaboost, LogitBoost, nb, and svmRadialWeights models were 0.996, 1.000, 1.000, 1.000, 0.988, and 0.988, respectively; the corresponding AUCs in the validation set were 0.786, 0.905, 0.905, 0.857, 0.952, and 0.952 (**Figures 3D**, **4**). Collectively, these results indicate that the three hub genes exhibit robust diagnostic potential, as the derived models show good performance in both the training and validation cohorts. Overall, diagnostic models constructed based on IL2RG, HOXD10, and TSPAN1 demonstrate excellent diagnostic efficacy for CASL.

### 3.4 Explanation of the diagnostic model based on SHAP

**Figure 5:**
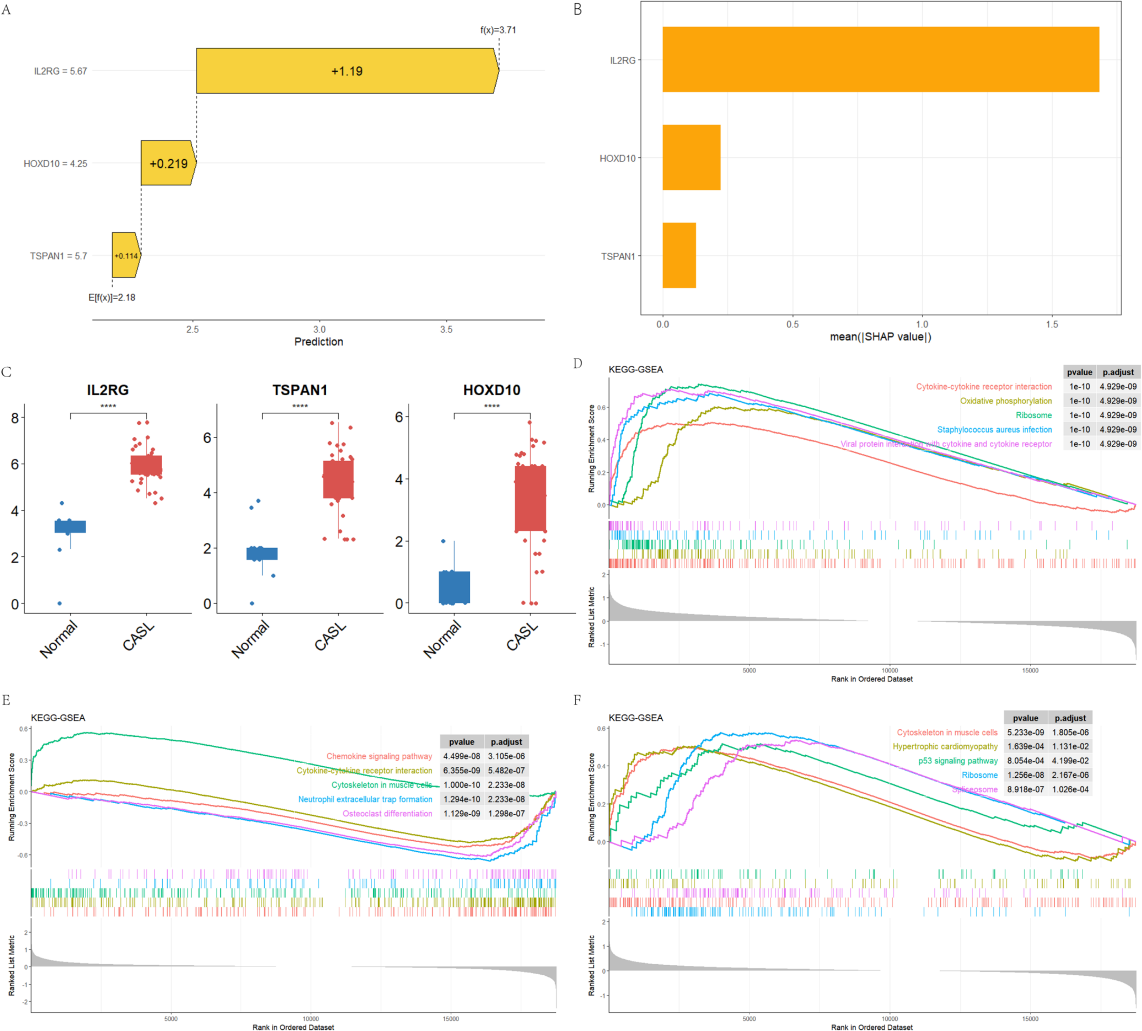
Hub gene expression, SHAP explanation, and functional enrichment. **(A)** The SHAP force plot for explaining how hub genes contribute to prediction outcomes in the model. **(B)** The SHAP importance plot illustrates the importance of three genes ranked from highest to lowest contribution. **(C)** Expression comparison of hub genes between CASL group and normal control group. **(D–F)** GSEA analyses of HOXD10, TSPAN1, and IL2RG.

SHAP is a widely used approach for model interpretability. Derived from Shapley values in game theory, SHAP provides a quantitative estimate of how each feature contributes to a model’s prediction. Positive SHAP values indicate that a feature increases the predicted probability of the outcome, whereas negative values indicate a decrease. To better characterize the contribution of the signature genes to the diagnostic model, SHAP analysis was performed on the trained XGBoost classifier. As presented in **Figures 5A, B**, the features ‘IL2RG=5.67’, ‘HOXD10 = 4.25’, and ‘TSPAN1 = 5.7’ positively contributed to the prediction of CASL, with SHAP values of 1.19, 0.219, and 0.114, respectively. These findings suggest that elevated expression levels of these genes increase the likelihood of a sample being classified as CASL. Notably, consistent with the SHAP interpretation, the expression levels of IL2RG, HOXD10, and TSPAN1 were significantly higher in the CASL group than in the normal group (*p* < 0.001, **Figure 5C**). Subgroup analysis showed that in the gynecological CASL subgroup, the expression levels of IL2RG, TSPAN1, and HOXD10 were significantly upregulated compared with the normal group, with all differences reaching high statistical significance (*p* < 0.001, **Supplementary Figure S1A**). The Naïve Bayes (nb) diagnostic model, constructed based on the three core genes, yielded an AUC of 0.875 (**Supplementary Figure S1B**), demonstrating robust discriminatory power for this specific subgroup. In the breast CASL subgroup, IL2RG, TSPAN1, and HOXD10 were similarly elevated to a highly significant degree (*p* < 0.01, **Supplementary Figure S1C**). The corresponding nb diagnostic model achieved an AUC of 0.75 (**Supplementary Figure S1D**), exhibiting favorable diagnostic efficacy in identifying this particular subpopulation. In conclusion, these data underscore the contributions of the three genes to model performance and demonstrate their potential involvement in CASL pathogenesis.

### 3.5 The GSEA analysis of diagnostic genes

To further investigate the biological functions associated with each diagnostic gene, GSEA was performed. HOXD10 was enriched in the chemokine signaling pathway, cytokine-cytokine receptor interaction, cytoskeleton in muscle cells, neutrophil extracellular trap formation, and osteoclast differentiation (*p* < 0.001, **Figure 5D**). TSPAN1 was closely linked to the cytoskeleton in muscle cells, hypertrophic cardiomyopathy, p53 signaling pathway, ribosome, and spliceosome (*p* < 0.05, **Figure 5E**). Meanwhile, IL2RG was significantly associated with the cytokine-cytokine receptor interaction, oxidative phosphorylation, ribosome, staphylococcus aureus infection, and viral protein interaction with cytokine and cytokine receptor (*p* < 0.001, **Figure 5F**).

### 3.6 The analysis of immune cell infiltration for CASL

**Figure 6:**
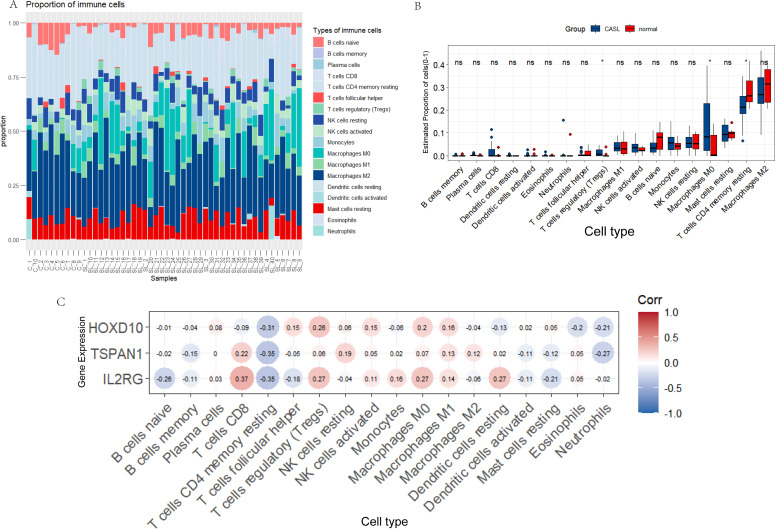
The composition of immune cells in the CASL group and normal control group. **(A)** The schematic diagram shows the expression levels of immune cells in each sample. **(B)** Differences in immune cell infiltration between the CASL group and the normal control group. **(C)** The correlation analysis between the hub genes and expressions of immune cell infiltration. The asterisk (*) indicates a statistically significant difference, with P < 0.05. The abbreviation "ns" represents not significant.

To explore the relationship between immune cells and CASL, we analyzed the relative infiltration abundance of immune cells using the CIBERSORT algorithm. As illustrated in **Figure 6A**, T cells and macrophages accounted for the largest proportions of infiltrating immune cells in both the normal and CASL groups. Coincidentally, compared with the normal group, regulatory T cells (Tregs) and M0 macrophages were more active in the CASL group (*p* < 0.05, **Figure 6B**). However, the levels of CD4 memory resting T cells were lower in the CASL group (*p* < 0.01, **Figure 6B**). No significant differences were observed in the other immune cell types between the two groups, which warrants further experimental validation.

To further validate the immune landscape and overcome the limitations of relative proportion analysis, the MCP-counter algorithm was employed to estimate the absolute abundance of immune and stromal cells. The cellular composition across all samples exhibited distinct distribution patterns (**Supplementary Figure S2A**). Consistent with the findings from CIBERSORT, the CASL group demonstrated a significant increase in the infiltration of several key immune populations. Notably, the estimated abundance of T cells and the monocytic lineage was markedly higher in the CASL group compared to the normal group (*p* < 0.0001, **Supplementary Figure S2B**). More importantly, MCP-counter facilitated the evaluation of stromal components that were not captured in the initial analysis. The results revealed that the absolute abundance of endothelial cells and fibroblasts was significantly elevated in CASL tissues (*p* < 0.001, **Supplementary Figure S2B**), suggesting a robust activation of lymphangiogenesis and fibrotic processes. Other cell types, including CD8+ T cells, cytotoxic lymphocytes, and myeloid dendritic cells, also showed varying degrees of enrichment in the CASL group, collectively contributing to a more complex and proinflammatory microenvironment in secondary lymphedema.

Subsequently, we assessed the correlations between the three diagnostic genes and immune cells. Interestingly, TSPAN1 and IL2RG were positively correlated with Tregs and M0 macrophages, whereas HOXD10, TSPAN1, and IL2RG were negatively correlated with CD4 memory resting T cells. This corresponded with the previous results. Additionally, TSPAN1 and IL2RG were positively correlated with CD8 T cells (**Figure 6C**). These findings demonstrate that T cells and macrophages may be involved in the pathogenic process of CASL.

### 3.7 Single-cell transcriptomic analysis

**Figure 7:**
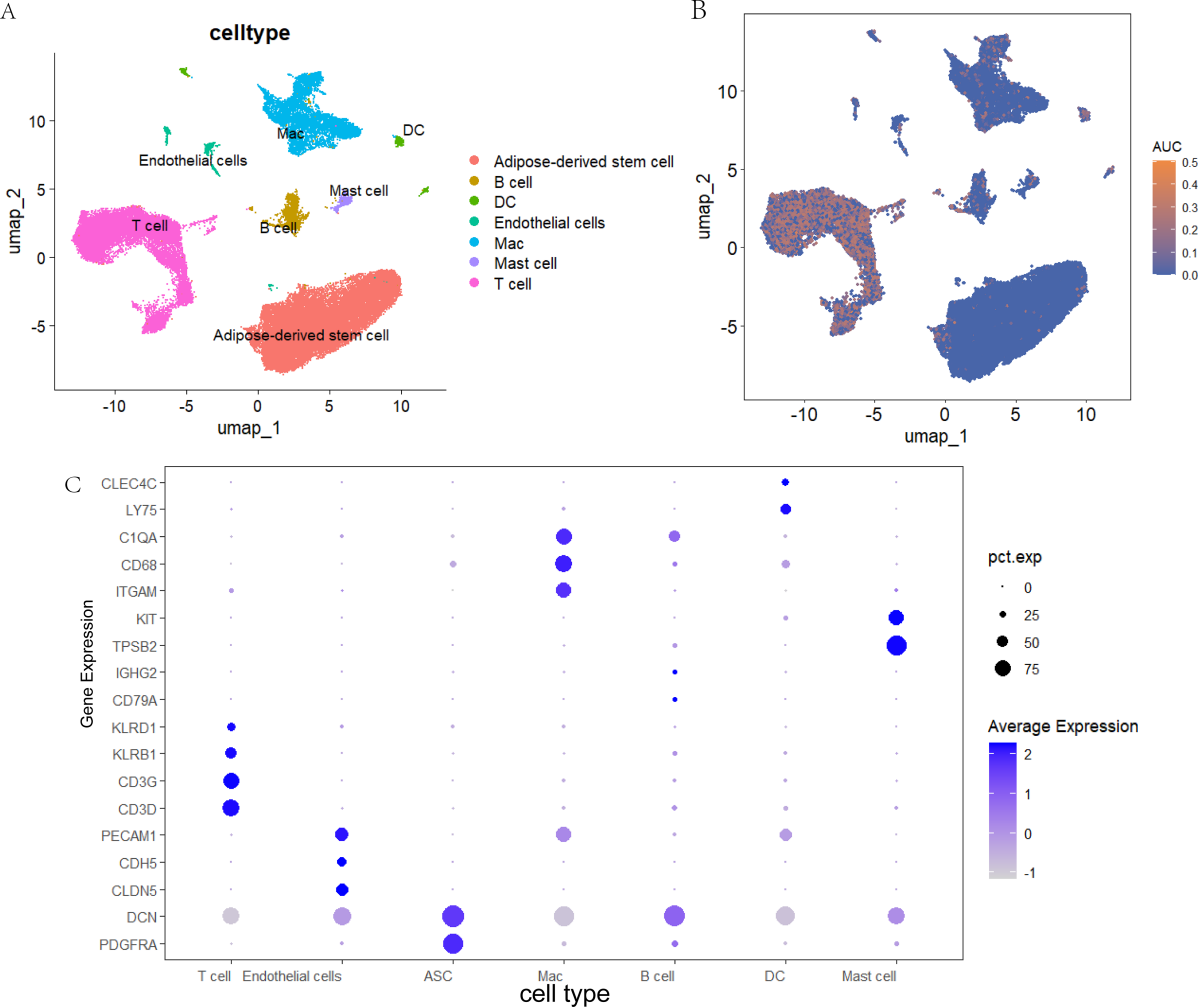
Single-cell transcriptomic analysis. **(A)** UMAP plot labeled by seven different cell types from CASL. **(B)** Analysis of hubgene in UMAP plot across different cells. **(C)** Dot plots showing differently expressed marker genes associated with CASL.

We integrated scRNA data from five CASL patients reported by Liu et al. After quality control, 40931 cells were retained and clustered into 15 clusters. Based on previously reported marker genes and CellMarker’s human cell markers, seven major cell types were identified (**Figure 7A**). AUCell scores were calculated using the three hub genes (IL2RG, HOXD10, and TSPAN1) to evaluate their activity across cell subtypes. The results indicated that these hub genes exhibited relatively higher activity in T cell clusters (**Figure 7B**). These marker genes were presented in **Figure 7C**: Adipose-derived stem cell (DCN, PDGFRA), B cell (CD79A, IGHG2), DC (CLEC4C, LY75), Endothelial cells (PECAM1, CDH5, CLDN5), Mac (C1QA, CD68, ITGAM), Mast cell (KIT, TPSB2), and T cell (CD3G, CD3D, KLRB1, KLRD1).

### 3.8 Results of RT-qPCR in human samples

**Figure 8:**
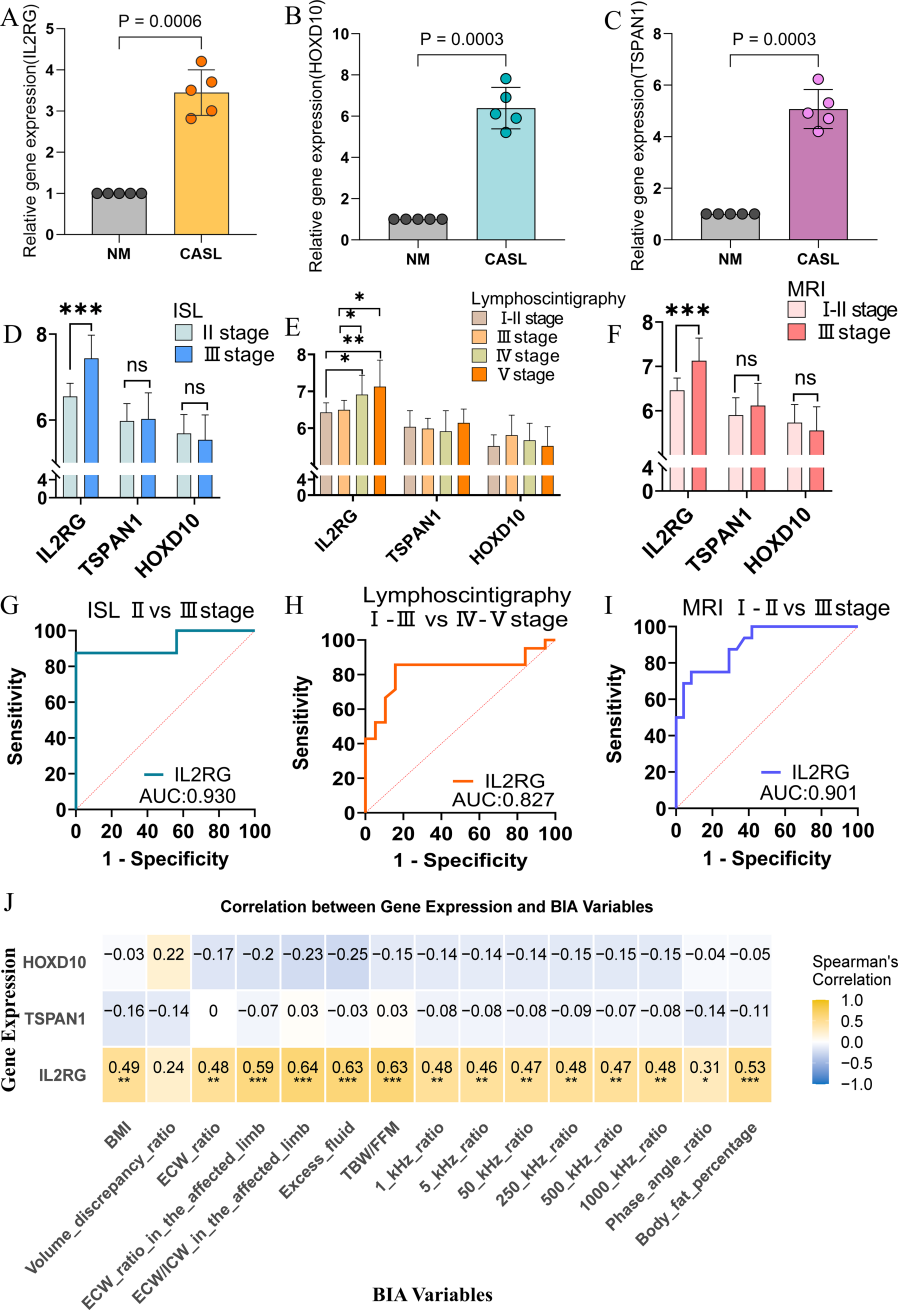
Validation of hub genes by RT-qPCR in human tissues and their association with clinical characteristics. **(A–C)** The expression levels of three characteristic genes in the CASL group and normal control group. **(D–F)** Hub gene expression in the three stage groups. **(G–I)** Diagnostic performance of IL2RG for differentiating advanced CASL stages across three classification systems. **(J)** Associations between hub genes and multiple parameters. *p < 0.05, **p < 0.01, ***p < 0.001.

We validated the expression levels of three core genes in adipose tissue from healthy individuals and CASL patients using RT-qPCR. IL2RG, HOXD10, and TSPAN1 were significantly upregulated in the CASL group compared to the control group (*p* < 0.001, **Figure 8A–C**). These experimental findings suggest that these genes may be potential pathogenic factors for CASL. This is consistent with our previous analysis.

### 3.9 Correlation between hub genes and clinical characteristics

Baseline clinical characteristics of the CASL cohort were summarized in **Table 1**. Compared with ISL stage II patients, those at stage III exhibited significantly higher BMI, MRI severity scores, and IL2RG expression, along with a shorter latency period. Furthermore, stage III patients displayed increased BIA parameters, including the volume discrepancy ratio, ECW ratio, excess fluid volume, and several others, as detailed in **Table 2**. Notably, IL2RG expression was significantly elevated in patients with advanced-stage CASL, as classified by ISL, MRI and lymphoscintigraphy (*p* < 0.05, **Figure 8D–F**). ROC curve analyses further demonstrated the ability of IL2RG to discriminate disease severity (**Figure 8G–I**). Additionally, IL2RG expression was positively correlated with multiple BIA parameters, including the ECW ratio, excess fluid and other relevant parameters (**Figure 8J**). These results collectively indicate a potential role for IL2RG in CASL progression.

**Table 1:** Baseline characteristics of CASL patients in the study.

Variable	ISL stage		*P*	Total
	II	III		
Subject number	32 (80.00%)	8 (20.00%)		40 (100.00%)
Age (years)	57.28 (7.02)	58.75 (9.78)	0.351	57.58 (7.53)
BMI (kg/m^2^)	25.91 (4.00)	31.91 (3.91)	<0.001	27.12 (4.62)
Lesion site				
Left upper limb	6 (18.75%)	0 (00.00%)	0.069	6 (15.00%)
Right upper limb	4 (12.50%)	0 (00.00%)		4 (10.00%)
Left lower limb	10 (31.25%)	7 (87.50%)		17 (42.50%)
Right lower limb	12 (37.50%)	1 (12.50%)		13 (32.50%)
Primary disease				
Breast cancer	10 (31.25%)	0 (00.00%)	0.169	10 (25.00%)
Endometrial cancer	5 (15.63%)	3 (37.50%)		8 (20.00%)
Cervical cancer	16 (50.00%)	5 (62.50%)		21 (52.50%)
Uterine Sarcoma	1 (3.12%)	0 (00.00%)		1 (2.50%)
Adjuvant therapy				
Radiotherapy	24 (75.00%)	8 (100.00%)	0.277	32 (80.00%)
Chemotherapy	25 (78.13%)	7 (87.50%)	0.921	32 (80.00%)
Latency period (months)	46.09 (64.75)	18.25 (31.72)	0.041	40.53 (60.33)
Disease duration (months)	59.56 (63.96)	49.50 (30.38)	0.695	57.55 (58.60)
Erysipelas	16 (50.00%)	5 (62.50%)	0.698	21 (52.50%)
Hypertension	6 (18.75%)	1 (12.50%)	1.000	7 (17.50%)
Hyperlipidemia	1 (3.13%)	1 (12.50%)	0.364	2 (5.00%)
Diabetes	4 (27.5%)	0 (00.00%)	0.566	4 (10.00%)
Coronary heart disease	0 (00.00%)	1 (12.50%)	0.200	1 (2.50%)
Lymphoscintigraphy stage				
I	1 (3.12%)	0 (00.00%)	0.056	1 (2.50%)
II	6 (18.75%)	1 (12.50%)		7 (17.50%)
III	11 (34.38%)	0 (00.00%)		11 (27.50%)
IV	12 (37.50%)	4 (50.00%)		16 (40.00%)
V	2 (6.25%)	3 (37.50%)		5 (12.50%)
MRI stage				
I	1 (3.12%)	0 (00.00%)	<0.001	1 (2.50%)
II	23 (71.88%)	0 (00.00%)		23 (57.50%)
III	8 (25.00%)	8 (100.00%)		16 (40.00%)
IL2RG	6.55 (0.31)	7.44 (0.54)	<0.001	6.73 (0.51)
TSPAN1	5.98 (0.41)	6.02 (0.61)	0.790	5.99 (0.44)
HOXD10	5.69 (0.44)	5.54 (0.58)	0.412	5.66 (0.47)

**Table 2:** Bioelectrical impedance analysis (BIA) parameters and clinically important data of CASL patients in the study.

Variable	ISL stage		*P*	Total
	II	III		
Volume discrepancy ratio (%) ^a^	30.54 (13.84)	53.71 (33.39)	0.004	35.17 (20.99)
ECW ratio (%) ^b^	38.21 (7.01)	41.84 (1.10)	<0.001	38.94 (6.43)
ECW ratio in the affected limb (%) ^c^	39.66 (7.01)	44.28 (1.32)	<0.001	40.58 (6.80)
ECW/ICW in the affected limb ^d^	0.70 (0.04)	0.80 (0.04)	<0.001	0.72 (0.06)
Excess fluid (L) ^e^	1.23 (0.99)	4.58 (1.72)	<0.001	1.90 (1.78)
TBW/FFM ^f^	74.02 (0.47)	74.84 (0.40)	<0.001	74.18 (0.56)
1 kHz ratio ^g^	1.58 (0.39)	2.52 (0.99)	0.032	1.77 (0.66)
5 kHz ratio	1.58 (0.38)	2.53 (0.99)	0.030	1.77 (0.66)
50 kHz ratio	1.53 (0.35)	2.46 (0.90)	0.022	1.71 (0.62)
250 kHz ratio	1.49 (0.32)	2.42 (0.86)	0.019	1.68 (0.60)
500 kHz ratio	1.48 (0.32)	2.36 (0.83)	0.002	1.65 (0.57)
1000 kHz ratio	1.49 (0.33)	2.42 (0.86)	0.002	1.68 (0.60)
Phase angle ratio ^h^	1.36 (0.23)	1.27 (0.24)	0.364	1.34 (0.23)
Body fat percentage (%)	36.76 (5.65)	44.04 (4.17)	0.002	38.21 (6.10)
Total protein	71.16 (5.41)	70.31 (6.71)	0.709	70.99 (5.61)
Albumin	41.37 (3.22)	39.10 (3.48)	0.124	40.92 (3.36)
Total cholesterol	5.49 (1.16)	5.23 (0.69)	0.544	5.44 (1.08)
Triglycerides	2.06 (1.14)	2.32 (1.13)	0.467	2.11 (1.13)
Glucose	6.48 (2.08)	6.06 (0.86)	0.660	6.40 (1.90)
HDL-C ^i^	1.24 (0.24)	1.29 (0.28)	0.582	1.25 (0.24)
LDL-C ^j^	3.64 (0.83)	3.29 (0.52)	0.267	3.57 (0.78)

**^a^**“Volume discrepancy ratio (%)” was defined as (volume of unaffected limb - volume of affected limb)/volume of unaffected limb * 100.

**^b^**“ECW ratio (%)” was defined as (extracellular water/total body water) * 100.

**^c^**“ECW ratio in the affected limb (%)” was defined as (extracellular water of affected limb/total body water of affected limb) * 100.

**^d^**“ECW/ICW in the affected limb” was defined as (extracellular water of affected limb/intracellular water of affected limb).

**^e^**“Excess fluid” was defined as the difference in total body water between the affected and unaffected limbs.

**^f^**“TBW/FFM” was defined as the ratio of total body water to fat-free mass.

**^g^**“1 kHz ratio” was defined as the impedance ratio at 1 kHz (unaffected/affected limb).

**^h^**“Phase angle ratio” was defined as the phase angle ratio (unaffected/affected limb).

**^i^**HDL-C, high-density lipoprotein cholesterol.

**^j^**LDL-C, low-density lipoprotein cholesterol.

## 4 Discussion

Lymphatic biology has attracted increasing attention from researchers. Historically, lymphatic disorders such as lymphedema were often been overlooked, highlighting the necessity for more comprehensive investigations across multiple dimensions. Especially for the study of CASL, it is important to reveal its molecular mechanism, diagnosis, and treatment to enhance patients’ quality of life (2). In this study, RNA-seq data from adipose tissue of 10 healthy individuals and 40 CASL patients were analyzed, leading to the identification of 365 DEGs. GO and KEGG enrichment analyses revealed that cytokine-cytokine receptor interactions and immune-related regulatory pathways were involved in CASL pathogenesis. Subsequently, two machine learning algorithms (LASSO and SVM-RFE) were applied to screen DEGs, and three core genes (IL2RG, HOXD10, and TSPAN1) were identified as potential diagnostic markers. To evaluate their diagnostic performance, seven machine learning algorithms were utilized to construct seven diagnostic models based on three core genes. At the same time, the accuracy of the seven models was evaluated by the ROC curve. The AUC values of the models demonstrated the superior diagnostic efficacy of these three signatures. To enhance interpretability, we further performed SHAP analysis on the XGBoost model. The SHAP values for IL2RG, HOXD10, and TSPAN1 were 1.19, 0.219, and 0.114, respectively, indicating positive contributions to CASL prediction. Specifically, higher values of these features were associated with an increased probability of being classified as CASL. Consistent with the SHAP interpretation, mRNA expression analyses showed that all three genes were significantly upregulated in the CASL group compared with controls, and these findings were further validated by RT-qPCR, supporting their potential roles as pathogenic contributors to CASL. Together, our findings support IL2RG, HOXD10, and TSPAN1 as promising molecular markers for CASL.

CASL is a complex multicellular response to specific cascade injuries characterized by inflammatory cell infiltration, dysregulated regional immune responses, adipose deposition, and fibrosis (2). Interleukin-2 receptor gamma chain (IL2RG)gene encodes the common gamma chain that is integral to the receptor complexes for several interleukins (IL-2, IL-4, IL-7, IL-9, IL-15, and IL-21) (23). Consequently, IL2RG participates in multiple signaling pathways that regulate the proliferation, differentiation, and survival of immune cells (24). Although IL-2 is not traditionally considered a direct driver of lymphedema, it may influence disease development through effects on immune cells. IL-2 is essential for the maintenance and functional expansion of regulatory T cells (Tregs) (25). Interestingly, the absence of Tregs exacerbates lymphedema in murine models (26). IL4 is a cytokine secreted by T helper cells, especially Th2 cells (27). IL2RG acts as its co-receptor, coordinating with the IL-4 receptor α chain (IL-4Rα) to mediate signal transmission (28). Common variants of IL4 have been significantly associated with the risk of lymphedema after breast cancer surgery. It may affect the development and progression of lymphedema through a variety of mechanisms. Firstly, IL-4 promotes the differentiation of CD4+ T cells into Th2 cells, which stimulates tissue fibrosis by producing pro-fibrotic cytokines like IL-13, thereby affecting lymphatic drainage (29). Moreover, M2 macrophages activated by IL-4 secrete various molecules, including transforming growth factor-β (TGF-β), interleukin-10 (IL-10), and CC chemokine ligand 18 (CCL18), that promote fibrosis by stimulating fibroblast proliferation and collagen synthesis (30). Unfortunately, there is currently no evidence to reveal that IL2RG directly affects CASL. Our study confirmed its high expression in CASL tissues, and GSEA revealed that IL2RG was mainly involved in cytokine-cytokine receptor interaction, aligning with the enrichment results of DEGs. In addition, immune cell infiltration analysis showed that IL2RG was positively correlated with T cells regulatory (Tregs) and Macrophages M0, while negatively correlating with T cells CD4 memory resting. In summary, IL2RG may influence the progression of CASL through cross-talk between immune cell infiltration, inflammation and fibrosis mediated by various cytokines. However, these findings require validation through extensive basic experimental research.

Lymphatic vessel growth and function are critical factors of lymphedema, and therapeutic lymphangiogenesis has been proposed as a promising treatment strategy (31). Homeobox D10 (HOXD10), a member of the homeobox gene family, plays key roles in embryonic development, including segment formation, limb development, and organ differentiation (32). As a transcription factor, HOXD10 regulates cellular processes such as proliferation, differentiation, and migration (33). Furthermore, HOXD10 is essential for regulating the growth and permeability of lymphatic vessels. A previous study showed that in lymphatic endothelial cells (LECs), HOXD10 was activated at an early time point after VEGFR-3 stimulation. On the one hand, HOXD10 overexpression promoted LECs migration and tubular morphogenesis, whereas HOXD10 knockdown produced the opposite effects. On the other hand, HOXD10 overexpression mediated the increase of LECs permeability induced by VEGF-C. Meanwhile, HOXD10 overexpression decreased the expression of genes associated with permeability and lymphangiogenesis (CDH5, CLDN5, TEK, and DLL4) and increased NOS3 expression in LECs. These findings suggest that elevated HOXD10 disrupts endothelial junctions and increases LECs permeability (34). Although the role of HOXD10 in CASL remains poorly understood, we observed higher HOXD10 expression in CASL tissues, and GSEA indicated enrichment in the chemokine signaling pathway and cytokine-cytokine receptor interaction, aligning with the DEG enrichment results. Additionally, HOXD10 expression was negatively correlated with resting memory CD4+ T cells. In conclusion, existing evidence supports a role for HOXD10 in LECs growth and function, and our study identifies it as a potential pathogenic factor in CASL. Nevertheless, the precise molecular mechanisms require further experimental validation.

Tetraspanin1 (TSPAN1) belongs to a family of four-transmembrane proteins that participate in many cellular activities. Four-transmembrane protein is a small hydrophobic transmembrane protein with a length of about 200–350 amino acids. It extends from the cell surface to 3–5 nm and interacts with other molecules on the cell membrane to form large molecular complexes, which participate in cell proliferation, migration, invasion, and signal transduction (35–37). Several studies have demonstrated that membrane microdomains formed by TSPAN1 with various proteins could affect the fibrosis phenotype mediated by epithelial-to-mesenchymal transition (EMT). Specifically, TSPAN1 was found to inhibit EMT in human pulmonary fibrosis by regulating the Smad2/3 and beta-catenin pathways (38). Furthermore, silencing TSPAN1 has been shown to reduce the polarization of CD4+ T cells into Th17 cells (39). Notably, dysdifferentiation of Th17 cells may exacerbate lymphedema. In early lymphedema, Th17 and Th1 cells could promote lymphangiogenesis by stimulating macrophages to generate VEGF-C, while in the late stage, they could inhibit lymphangiogenesis (40). Although the role of TSPAN1 in CASL has not been reported, based on the above studies, we speculate that it may mediate the progression of CASL through immune and fibrotic mechanisms. Importantly, our research confirmed the high expression of TSPAN1 in CASL tissues and suggested its involvement in cytoskeletal processes in muscle cells and the p53 signaling pathway. Immune infiltration analysis indicated that TSPAN1 was positively associated with T cells regulatory (Tregs), CD8+T cell, and Macrophages M0, and negatively associated with T cells CD4 memory resting. Nonetheless, the effect of TSPAN1 on CASL still needs to be further revealed in more experiments.

The pathogenesis of CASL is intrinsically linked to local immune responses. Development of CASL is typically accompanied by tissue injury and inflammatory cascades that trigger the activation and infiltration of immune cells. Previous studies have established that macrophages and T cells accumulate significantly at lymphedema sites, where they secrete pro-inflammatory cytokines and chemokines that exacerbate local inflammation and fibrosis (9, 26, 40). In the present study, the CIBERSORT algorithm was utilized to characterize the immune cell infiltration landscape. T cells and macrophages were the predominant cell types in both the normal and CASL groups, which is consistent with prior findings. Interestingly, the proportions of T cells regulatory (Tregs) and Macrophages M0 were higher in the CASL group than in the normal group, whereas T cells CD4 memory resting were significantly lower. Subsequent correlation analysis between the three diagnostic genes and immune cell subsets revealed that TSPAN1 and IL2RG were positively correlated with Tregs and M0 macrophages. Furthermore, HOXD10, TSPAN1, and IL2RG exhibited negative correlations with T cells CD4 memory resting, while TSPAN1 and IL2RG showed positive associations with CD8+ T cells. Concordantly, scRNA-seq analysis demonstrated that significant enrichment of IL2RG, HOXD10, and TSPAN1 transcripts in T cell clusters. These results further demonstrate that T cells and macrophages are involved in the pathogenesis of CASL.

To further validate these findings and overcome the limitations of relative proportion analysis, the MCP-counter algorithm was employed to estimate the absolute abundance of immune and stromal populations. This analysis not only corroborated the enrichment of T cells and monocytic lineages in CASL tissues but also highlighted a profound expansion of the stromal compartment. Specifically, the significant elevation of fibroblasts and endothelial cells in the CASL group suggests a robust activation of fibrogenesis and microvascular remodeling. The concurrent increase in both monocytic cells and fibroblasts reinforces the hypothesis that a persistent inflammatory-fibrotic axis drives the progressive tissue fibrosis characteristic of secondary lymphedema. Furthermore, the higher abundance of endothelial cells may reflect compensatory but dysfunctional lymphangiogenesis in response to chronic lymphatic stasis. These results further demonstrate that the coordinated interplay between T cells, macrophages, and stromal components underpins the pathogenesis of CASL.

However, several limitations of this study should be acknowledged. Primarily, our research was conducted as a single-center investigation with a relatively modest sample size. Although our cohort surpassed those of previously reported studies, the current scale remains limited, which may constrain statistical power and limit the generalizability of our findings. Future multi-center studies with larger populations are necessary to validate and extend our findings. Furthermore, while cross-validation was performed using two distinct algorithms to characterize immune cell infiltration in CASL, these in silico findings warrant further experimental verification. Lastly, this study identified correlations between IL2RG, HOXD10, TSPAN1, and CASL exclusively through bioinformatic analyses and expression validation. Therefore, comprehensive *in vivo* and *in vitro* experiments are still required to confirm the underlying causal relationships and molecular mechanisms.

## 5 Conclusions

In the present study, by integrating RNA-seq data with machine learning algorithms, IL2RG, HOXD10, and TSPAN1 were identified as key upregulated molecular markers in CASL. These markers were subsequently utilized to construct interpretable diagnostic models that exhibited excellent diagnostic performance. Simultaneously, our analysis revealed that the regulation of cytokine-cytokine receptor interaction and immune pathways may be the potential pathogenesis of CASL. Furthermore, T cells and macrophages appeared to be involved in the progression of CASL. In addition, our findings were preliminarily validated with clinical specimens. Ultimately, we hope that our study will provide meaningful contributions to the diagnosis and treatment of CASL based on molecular markers.

## Data Availability

The datasets presented in this study can be found in online repositories. The names of the repository/repositories and accession number(s) can be found below: https://bigd.big.ac.cn/gsa-human/browse/HRA013043, HRA013043.
