# Correction: Uncovering potential molecular biomarkers for cancer-associated secondary lymphedema through integrated analyses of RNA-sequencing, machine learning, and clinical data

**DOI:** 10.3389/fonc.2026.1894847

**Published:** 2026-07-09

**Authors:** Hao Dong, Jianliang Miao, Zhong Liu, Yuguang Sun, Peilin Li, Song Xia, Wenbin Shen

**Affiliations:** 1 Capital Medical University Affiliated Beijing Shijitan Hospital, Beijing, China; 2 The First Affiliated Hospital of Dalian Medical University, Dalian, China

**Keywords:** immune infiltration, machine learning, ScRNA, secondary lymphedema, Shap

**Supplementary Data Sheet 1** was erroneously published with the original version of this paper. There was a mistake in the GAPDH primer sequence in Table S1. The corrected file has been published.

The original version of this article has been updated.

