# Supplementary material for: Uncovering potential molecular biomarkers for cancer-associated secondary lymphedema through integrated analyses of RNA-sequencing, machine learning, and clinical data
Source: Front Oncol. 2026 Feb 13;16:1760040. doi: 10.3389/fonc.2026.1760040 (PMC12945810; doi:10.3389/fonc.2026.1760040)
Supplement: Supplementary file 1 [file DataSheet1.docx]

**Table S1** All primer sequences used in this study.

| Gene | Forward primer (5′-3′) | Reverse primer (5′-3′) |
| --- | --- | --- |
| GAPDH | GATTTGGTCGTATTGGGCGC | TTCCCGTTCTCAGCCTTGAC |
| IL-2RG | ATTTCTGGCTGGAACGGACG | GCCAGTCCCTTAGACACACC |
| HOXD10 | GACATGGGGACCTATGGAATGC | TGGTGGTTCACTTCTCTTTTGG |
| TSPAN1 | ACGACCAAAAAGTAGAGGGTTG | CATGGACACAATCATGGCAGC |

**Table S2** Hyperparameter configurations of the machine learning algorithms employed in this study.

| Algorithm | Key hyperparameter configurations |
| --- | --- |
| Naïve Bayes (nb) | Implemented using the default Gaussian variant, which assumes that features follow a normal distribution. |
| svmRadialWeights | Implemented with a Radial Basis Function (RBF) kernel (kernel = 'rbf'). The regularization parameter C was set to 1.0, and the kernel coefficient gamma was set to 'scale'. Class weights were balanced during training (class_weight = 'balanced'). |
| Random Forest (rf) | The ensemble consisted of 500 decision trees (n_estimators=500), with no constraint on the maximum depth of individual trees (max_depth=None). The minimum number of samples required to split an internal node was set to 2 (min_samples_split = 2), while the minimum number of samples required at a leaf node was set to 1 (min_samples_leaf = 1). The number of features to consider for the best split was determined by the square root of the total features (max_features = 'sqrt'). |
| Kernel k-Nearest Neighbors (kknn) | The number of neighbors was set to 5 (n_neighbors = 5), using distance-based weighting (weights = 'distance'). The distance metric was Minkowski with p = 2 (equivalent to Euclidean distance), and the kernel function was set to 'optimal'. |
| AdaBoost (adaboost)  LogitBoost | The model was run for 50 boosting iterations (n_estimators = 50) with a learning rate of 1.0 (learning_rate = 1.0) and utilized the SAMME discrete boosting algorithm (algorithm = 'SAMME').  Similarly configured for 50 iterations (n_estimators=50) with a learning rate of 1.0 (learning_rate=1.0). |


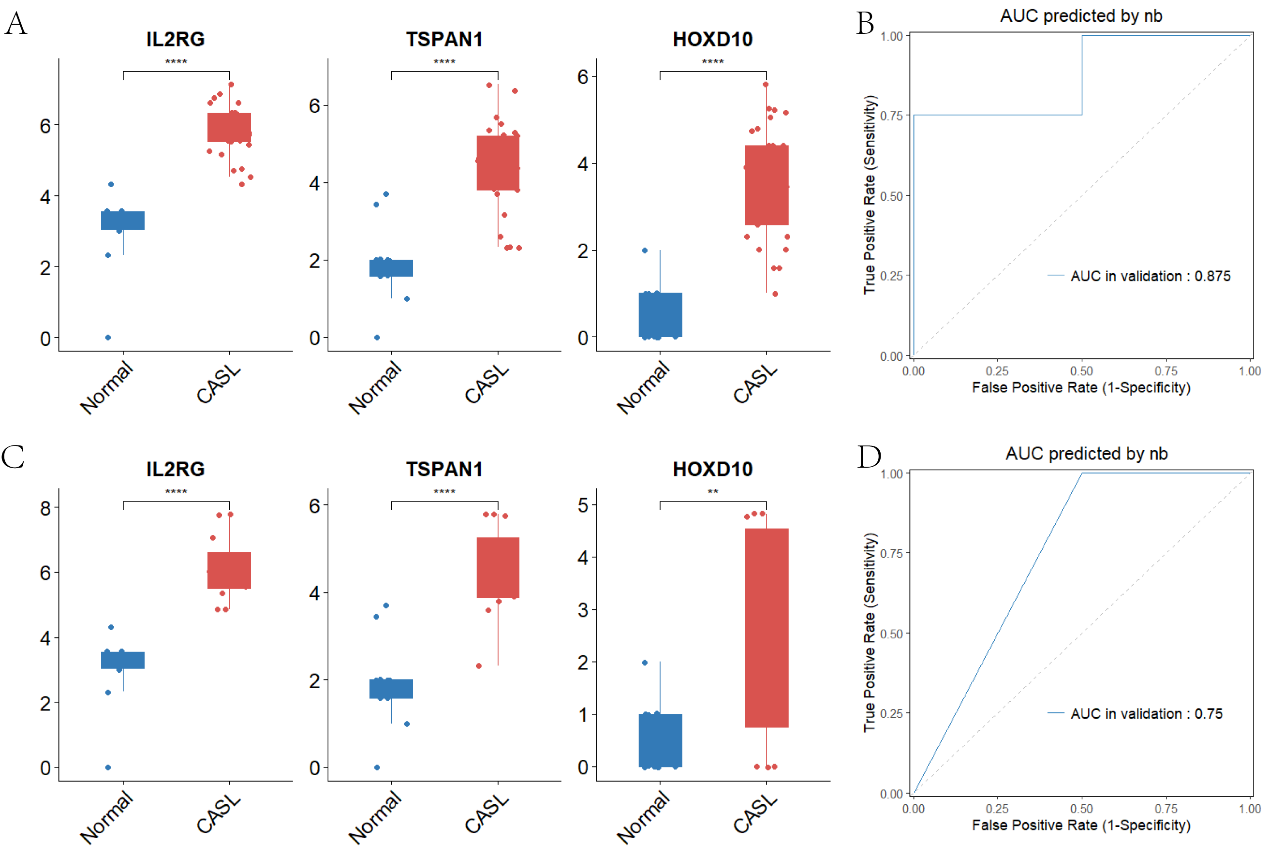


**Figure S1** Validation of core gene expression and diagnostic model performance in CASL subgroups stratified by primary cancer type. (A) Expression levels of the three core genes (IL2RG, TSPAN1, and HOXD10) in patients with gynecological CASL compared to the controls. (B)ROC curve demonstrating the diagnostic efficacy of the model constructed from the three core genes for discriminating gynecological CASL patients from controls in the dataset. (C) Expression levels of the same three core genes in patients with breast CASL compared to the controls. (D) ROC curve showing the diagnostic performance of the core-gene-based model in the breast CASL subgroup.


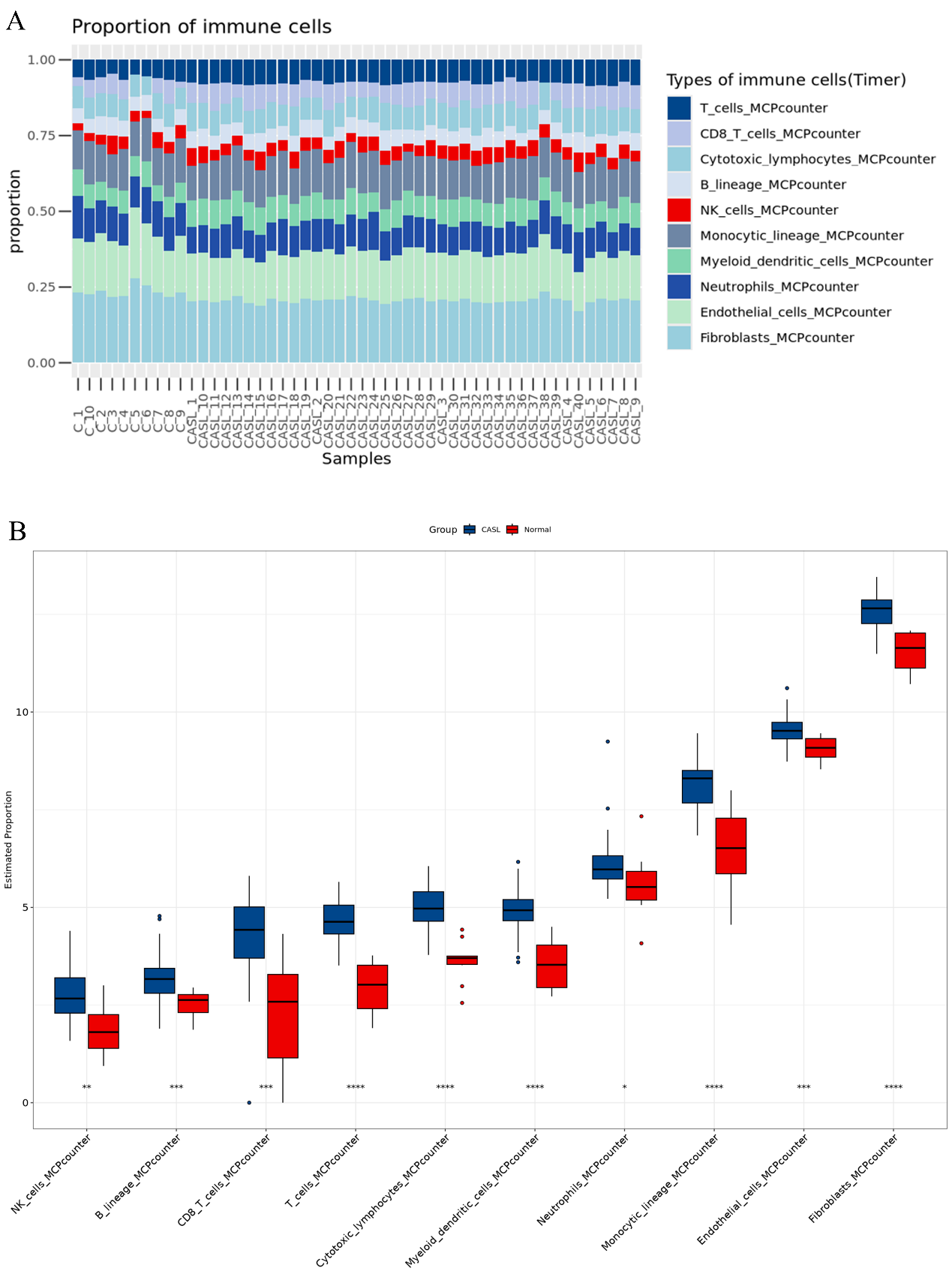


**Figure S2** Assessment of the immune and stromal microenvironment using the MCP-counter algorithm. (A) Stacked area plot illustrating the landscape of cell infiltration across samples. The distribution and relative proportions of 10 immune and stromal cell types (e.g., T cells, CD8 T cells, Endothelial cells, and Fibroblasts) were quantified. The y-axis represents the estimated proportion (0.00–1.00), and the x-axis denotes individual samples. (B) Boxplot comparing the estimated cell abundance between groups. Comparisons of cell populations were performed between the CASL group (blue) and the Normal group (red). **p* < 0.05, ** *p* < 0.01, *** *p* < 0.001, **** *p* < 0.0001.
